# TLR Signaling Paralyzes Monocyte Chemotaxis through Synergized Effects of p38 MAPK and Global Rap-1 Activation

**DOI:** 10.1371/journal.pone.0030404

**Published:** 2012-02-09

**Authors:** Ling Yi, Prabha Chandrasekaran, Sundararajan Venkatesan

**Affiliations:** Molecular Cell Biology Unit, Laboratory of Molecular Immunology, National Institute of Allergy and Infectious Diseases and National Institutes of Health, Bethesda, Maryland, United States of America; University of Delhi, India

## Abstract

Toll-like receptors (TLRs) that recognize pathogen associated molecular patterns and chemoattractant receptors (CKRs) that orchestrate leukocyte migration to infected tissue are two arms of host innate immunity. Although TLR signaling induces synthesis and secretion of proinflammatory cytokines and chemokines, which recruit leukocytes, many studies have reported the paradoxical observation that TLR stimulation inhibits leukocyte chemotaxis *in vitro* and impairs their recruitment to tissues during sepsis. There is consensus that physical loss of chemokine receptor (CKR) at the RNA or protein level or receptor usage switching are the mechanisms underlying this effect. We show here that a brief (<15 min) stimulation with LPS (lipopolysaccharide) at ∼0.2 ng/ml inhibited chemotactic response from CCR2, CXCR4 and FPR receptors in monocytes without downmodulation of receptors. A 3 min LPS pre-treatment abolished the polarized accumulation of F-actin, integrins and PIP_3_ (phosphatidylinositol-3,4,5-trisphosphate) in response to chemokines in monocytes, but not in polymorphonuclear neutrophils (PMNs). If chemoattractants were added before or simultaneously with LPS, chemotactic polarization was preserved. LPS did not alter the initial G-protein signaling, or endocytosis kinetics of agonist-occupied chemoattractant receptors (CKRs). The chemotaxis arrest did not result from downmodulation of receptors or from inordinate increase in adhesion. LPS induced rapid p38 MAPK activation, global redistribution of activated Rap1 (Ras-proximate-1 or Ras-related protein 1) GTPase and Rap1GEF (guanylate exchange factor) Epac1 (exchange proteins activated by cyclic AMP) and disruption of intracellular gradient. Co-inhibition of p38 MAPK and Rap1 GTPase reversed the LPS induced breakdown of chemotaxis suggesting that LPS effect requires the combined function of p38 MAPK and Rap1 GTPase.

## Introduction

Cells of myelomonocytic lineage constitute the first lines of defense against pathogens. Of these, the short-lived, free-roaming neutrophils are the primary sentinels that respond to intrinsic and extrinsic chemical and environmental cues from the inflammatory foci, and set up robust anti-microbial effector functions. Monocytes and monocyte-derived macrophages subserve other functions including, but not limited to phagocytosis, antigen presentation, and initiation of acquired immunity.

Leukocytes are endowed with a diverse family of G-protein coupled receptors (GPCRs) that sense chemoattractants and regulate directed cell migration in the immune system [1]. During chemotaxis, which is governed by spatially restricted integration of diverse signaling pathways that lead to cell polarity [2], leukocytes change rapidly from a roughly spherical to a polarized morphology with distinct leading and trailing edges and F-actin accumulation at the front [3].

Toll-like receptor (TLR) family members recognize microbial products to usher in innate immune response and bridge the innate and acquired immune response to pathogens Of the TLRs, TLR4 forms hetero- and homo-dimers at the cell surface and is the sole receptor for lipoplysaccharide (LPS). TLR signaling stimulates various transcriptional pathways, which prime innate immune cells against pathogens by facilitating pro-inflammatory cytokine and chemokine secretion [4].

Both positive and negative effects of TLR ligands on neutrophil chemotaxis have been documented. Besides being recognized as a surrogate chemoattractant by neutrophils [5]; LPS treatment enhanced neutrophil chemotaxis through increased expression (and secretion) of chemokines and cognate receptors [6]; induction of MMP-8 cleavage of LIX chemokine to enhance CXCR2 binding [7]; or downregulation of GRK2 and GRK5 (G protein-coupled receptor kinase 2 or 5) mRNAs thus decreasing CXCR2 desensitization [8]. In contrast, septic neutrophils or control cells treated with cytokines plus LPS or LTA were impaired for chemotaxis through enhanced GRK2 and GRK5 expression [9] or down-modulation of CXCR1/2 [10]. LPS also inhibited migration towards endogenous chemokines through p38 MAPK and concomitant inhibition of PI3K (phosphatidylinositol-3-phosphate kinase) [11].

TLR signaling also downregulates CKRs on monocytes. LPS at 100 ng/ml for 1 h decreased the steady state levels of CCR2 and to a lesser extent, CCR1 and CCR5 mRNA [12], [13] and promoted tyrosine kinase mediated serine protease degradation of CCR2 [14]; LPS at 10 µg/ml enhanced chemokine secretion, which downregulated cognate receptors CCR1 and CCR2 through autocrine pathway [15]; 20 hr treatment with Pam_3_CSK_4_ at 50 ng/ml induced selective reduction in chemokine receptor transcript levels [16]; while treatment with LTA at 10 µg/ml for 1 h downregulated CCR1, 2 and 5 on human monocytes by recruiting the endocytic machinery of agonist mediated downmodulation [17].

LPS at 1 µg/ml increased adherence of THP-1 monocytes through PI3-K mediated LFA-1 (leukocyte function-associated antigen-1 or integrin αLβ2) activation [18] or by Rap1 GTPase regulated Mac-1 (macrophage-1 antigen or integrin αMβ2) activation [19]; or by actin reorganization by phosphorylated tyrosine kinase, Pyk2 and paxillin [20]. More recently it was shown that while LPS at 1 µg/ml for 15 min increased integrin activation and matrix adhesion and a corresponding decrease in TEM under static conditions, it did not alter monocyte adhesion or migration under conditions of physiological flow [21].

As extensive and varied as the above observations are, they represent results of TLR signaling at high ligand inputs over prolonged periods when the full gamut of TLR signaling is in play. Here, we have evaluated the effects of short-term (15 min) treatment of pro-inflammatory leukocytes with limiting amounts of TLR agonists. We show that LPS (2 ng/ml), induced immediate cell spreading and chemotactic arrest in primary human monocytes, but not in PMNs and myelomonocytic cell line U-937. Chemotactic arrest resulted from rapid global induction of PIP_3_ production, which primed p38 MAPK and Rap1 signaling. These events led to symmetric LFA-1 and Mac-1 activation and global F-actin distribution, both of which combined to increase adhesion and inhibit chemotaxis. Combined inhibiton of p38 MAPK and Rap1 GTPase restored chemotaxis in LPS treated cells.

## Results

### LPS and other TLR2/4 ligands induced cell spreading and inhibited chemotactic polarization and migration of primary human monocytes

**Figure 1:**
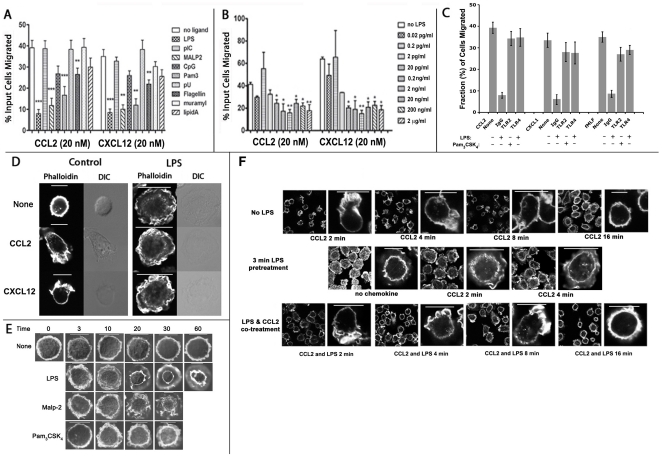
LPS treated human monocytes displayed flattened morphology and are inhibited for chemotaxis. **A**) Relative chemotactic inhibition by different TLR ligands. Primary human monocytes were treated with the indicated TLR ligands exactly as described under **Methods** and allowed to migrate towards 20 nM CCL2 or CXCL12 in the Trans-well chamber. Data are plotted as histograms with error bars (n = 3; **, p<0.03; ***, p<0.01). **B**) LPS dose response of chemotactic inhibition of monocytes. Monocytes (5×10^5^) were pretreated for 15 min with the various concentrations of LPS, washed 3 X and placed in the upper wells of 5.0 µm Trans-well and allowed to migrate towards 20 nM CCL2 or CXCL12 in the bottom wells. Relative fraction (%) of input cells that migrated to the bottom well is plotted in the histogram with error bar (n = 3; *, p<0.03, **, p<0.04). **C**) Chemotaxis of monocytes towards CCL2, CXCL12 or fMLF after preincubation with TLR2, TLR4 ligands in the presence of TLR2, TLR4 antibodies or isotype IgG control. Relative fraction (%) of migrated cells is plotted in the histogram with error bars. **D**) LPS pretreatment abolished chemokine induced F-actin polarizarion. Fresh monocytes on cover slips were treated with or without LPS (2 ng/ml) for 15 min at 37°C prior to 2 min stimulation with 20 nM CCL2 or CXCL12. Cells were fixed and stained with Alexa-488 conjugated phalloidin and examined by fluorescent microscopy and interference optics (DIC). **E**) Time course of morphological change in monocytes induced by various TLR ligands (LPS, 2 ng/ml; MALP-2, 200 ng/ml; and Pam_3_CSK_4_, 50 ng/ml). Cells were fixed and stained for F-actin as above. **F**) F-actin polarization was preserved in monocytes stimulated with 20 nM CCL2 prior to (top row) or simultaneously (bottom row) with LPS at 2 ng/ml. The middle row shows results with cells pretreated with LPS for 3 min before CCL2. Figures in **D**, **E** and **F** represent three independent experiments using monocytes from different donors.

LPS and other TLR ligands caused severe inhibition of chemotactic potential of monocytes towards CCL2, CXCL12 (Figure 1 A) or fMLF (not shown) in the Trans-well assay. To determine if this characteristic was limited to some TLR ligands, we tested monocyte chemotaxis after treatment with TLR4 specific LPS, TLR1/2 heterodimer ligand MALP2 (macrophage-activating lipopeptide-2), TLR3 targeting poly I∶C, TLR2/6 specific tripalmitoylated lipopeptide, Pam_3_CSK_4_; TLR5 ligand flagellin, poly(U) that binds TLR7 or 8, TLR9 ligand CpG ODN (oligo-deoxynucleotide), lipid A (TLR4) and a NOD2 (nucleotide-binding oligomerization domain) ligand, muramyl dipeptide. Chemotactic inhibition was observed only with ligands for TLRs at the cell surface like LPS, MALP2, Pam_3_CSK_4_ and to a less extent flagellin (Figure 1 A).

Substantial inhibition of monocyte chemotaxis towards CCL2 or CXCL12 was observed with LPS around 0.2 ng/ml (Figure 1 B). A 3–10 min treatment with LPS or MALP2 inhibited monocyte chemotaxis towards CCL2, CXCL12 or fMLF. While the inhibitory effect of a 3 min LPS treatment was irreversible, a longer exposure with MALP2 (10–30 min) was necessary to induce a recalcitrant state (Figure S1). Preincubation with TLR2 or TLR4 antibodies prevented chemotactic inhibition by cognate TLR agonists (Figure 1 C).

Within 2 min of addition of 20 nM CCL2 or CXL12, monocytes displayed polarization and assembly of F-actin polymers at the leading edge. However, a 15 min LPS pretreatment abolished this response; instead the cells acquired flattened morphology with circumferential distribution of F-actin (Figure 1 D, Control vs. LPS). To evaluate the immediate effects of TLR agonists, we adopted the cell polarization assay to examine the time course of chemotactic polarization. LPS, MALP-2 and Pam_3_CSK_4_ induced cell spreading with circumferential F-actin within 3–10 min (Figure 1 E).

To evaluate the primacy of LPS induced arrest over chemokine induced polarization, and *vice versa*, we treated monocytes with LPS either simultaneously with or shortly after the chemokine stimulation. As illustrated by Figure 1 F (top row), human monocyte polarized within 2 min after CCL2 addition, slowly decaying to ground state by 16 min. Adding LPS at the same time as CCL2 did not impair this polarization (Figure 1F, bottom row). However, adding LPS 3 min prior to CCL2 inhibited polarization (Figure 1F, middle row). Preincubation with TLR2 or TLR4 antibodies prevented chemotactic inhibition by cognate TLR agonists (not shown).

### Neutrophil polarization or chemotaxis was not inhibited by short-term LPS treatment

**Figure 2:**
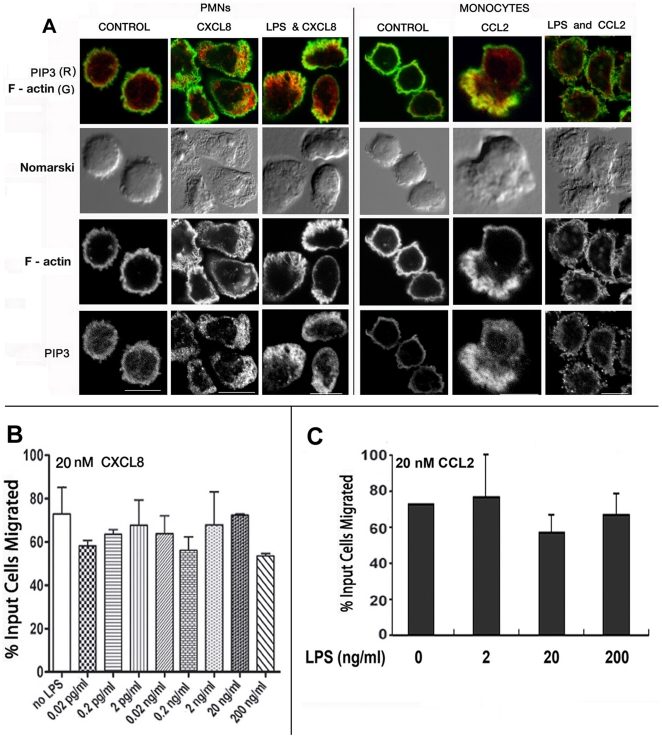
Differential effects of LPS on neutrophil and monocyte chemotaxis. **A**) LPS pre-treatment affects the polarization of CCL2 treated monocytes but not CXCL8 treated neutrophils. Human monocytes and neutrophils were plated on cover slips and treated with or without LPS (2 ng/ml) for 10 min at 37°C. After washing, cells were treated with CXCL8 (PMNs) or CCL2 (MONOCYTES) at 20 nM at 37°C for 2 min. Cells were then fixed, permeabilized and stained with Alexa568- phalloidin and FITC mAb against PIP_3_ and imaged by fluorescence and DIC microscopy. **B**) LPS did not significantly inhibit neutrophil chemotaxis. Human neutrophils (1×10^6^ cells in triplicate) were treated with various concentrations of LPS in 100 µl RPMI with 5% FBS for 10 min at 37°C. After washing, cells were suspended in 100 µl RPMI with 1% FBS and added to the upper chambers of 5.0 µm Trans-well (Corning Inc.), with or without 20 nm CXCL8 or fMLF (not shown) in the bottom well, and incubated for 2 h at 37°C. Migrated cells were collected and counted, as described under **Methods**. Fraction (%) of migrated input cells are presented in the histograms (with error bars), n = 3. **C**) LPS treatment at 20 ng/ml induced little or no inhibition of of U937 myelmonocytic cell chemotaxis towards 20 nM CCL2, n = 3.

We compared the LPS effect on polarization of PMNs vs. monocytes towards their respective chemokines, CXCL8 or CCL2. Persistent F-actin polarity and other cytoskeletal dynamics in response to chemoattractants depend on polarized PI3K activation leading to PIP_3_ accumulation at the leading edge. LPS treated or untreated PMNs were stimulated with 20 nM CXCL8 for 2 min, fixed and permeabilized and stained with fluorescent phalloidin and anti-PIP_3_ mAb. CXCL8 induced marked F-actin polarization with PIP_3_ at the leading edge whether or not PMNs were treated with LPS (Figure 2 A). However, this was not the case for LPS treated monocytes, which lost CCL2 induced F-actin and PIP_3_ polarization at the leading edge.

LPS at 0.02 pg/ml induced a mild inhibition of neutrophil migration towards CXCL8, but there was no dose (LPS 0.02 pg–0.2 µg/ml) dependent decrease in chemotaxis (Figure 2 B). Neutrophils exposed to different TLR ligands at 20 ng/ml or less for ∼20 min were not significantly inhibited for migration towards fMLF or CXCL8 other than the potent inhibitory effect of MALP-2 (data not shown). Consistent with a previous report [10], longer (1–2 h) LPS treatment at >200 ng/ml inhibited neutrohil chemotaxis significantly (not shown), probably resulting from down-modulation of chemokine receptor CXCR1/2 [10]. Chemotaxis of human myelomonocytic cell line U937 towards CCL2 was also not significantly inhibited by LPS treatment over a range of 2–200 ng/ml (Figure 2 C).

### LPS treatment did not alter the steady state levels of CKRs, their G-protein signaling potential or enhance endocytosis of agonist occupied receptors

**Figure 3:**
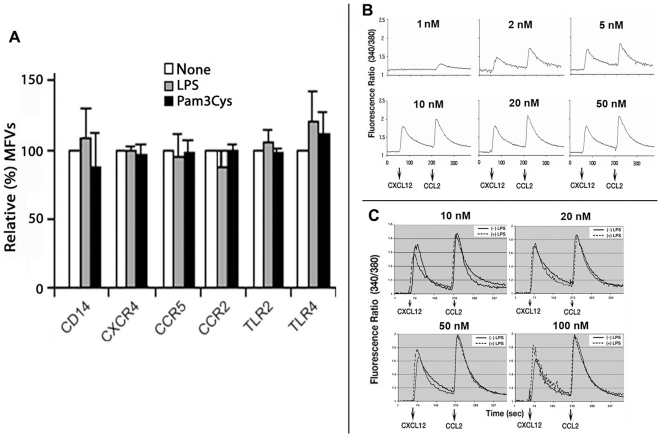
Short term stimulation of monocytes with TLR2 or TLR4 ligand did not significantly modulate cell surface expression of selected immune cell receptors and LPS treatment did not alter the G-protein signaling potential (as measured by intracellular Ca^2+^ flux) to CXCL12 or CCL2 treatment(s). **A**) Monocytes were treated with LPS (2 ng/ml) or Pam_3_CSK_4_ (50 ng/ml) for 15 min before staining with fluorescent mAbs against the indicated immune cell receptors. Receptor densities were measured by flow cytometry and the MFVs in stimulated cells are expressed relative (%) to untreated cells as histograms (with error bars, n = 3). **B**) Agonist dose response of intracellular Ca^2+^ flux to sequential stimulation with CXCL12 and CCL2 in monocytes. **C**) Ca^2+^ flux profiles of LPS (2 ng/ml, for 15 min after calcium dye loading) treated (interrupted line) and untreated monocytes to sequential stimulation with increasing concentrations of CXCL12 and CCL2. Plots represent results using monocytes from four donors.

Inhibition of chemotaxis by LPS treatment might reflect downregulation or degradation of CKRs or their respective mRNAs. If TLR stimulated cells secreted chemokines, they might desensitize cognate receptors in an autocrine manner. However, this was unlikely since pro-inflammatory cyto- and chemokine secretion occurred only after >2 h treatment with various TLR ligands including LPS at 100 ng/ml or more (Figure S2 A and B). Consistent with this observation, there was no loss in the steady-state levels of many different immune cell receptors under the limited LPS treatment conditions used here (Figure 3 A).

We inquired whether LPS treatment compromised chemoattractant signaling or accelerated endocytosis of agonist occupied receptors. We examined the earliest step(s) in GPCR signaling, namely Gβγ dissociation from the agonist occupied receptor. Gβγ dissociation was measured indirectly by monitoring intracellular Ca^++^ flux from ER stores mediated by IP_3_ from PIP_2_ hydrolysis by Gβγ activated PLC-β. 2×10**^6^** monocytes were preloaded with the fluorescent Ca^++^ indicator FURA-2 and then treated with LPS or not. We compared the agonist dose response profiles of Ca^++^ flux in control and LPS treated cells after sequential stimulation with CCL2 and CXCL12. LPS treated monocytes did not exhibit any differences in the kinetics or the magnitude of calcium flux (Figure 3B), with the exception of one donor whose monocytes were attenuated by LPS for calcium flux from CXCR4 by 40% at the lowest (10 nM) CXCL12 concentration.

LPS treatment has been demonstrated to modulate the cell surface levels of many receptors through facilitated endocytosis by direct binding [17], enhanced constitutive endocytosis or recruiting alternative endogenous agonist(s). To obtain a quantitative measure of receptor clearance in the control vs. LPS treated cells, we compared the dose-response and rate curves of agonist-mediated receptor internalization from the LPS vs. untreated monocytes. LPS treatment inhibited the magnitude of CCR2 and CXCR4 internalization at the higher concentrations of chemokine. As illustrated in Figure S3 B, the EC_50_ was 3–4 fold higher and *t_1/2_* values two fold more in LPS treated cells for the two agonist∶receptor combinations.

### LPS induced the phosphorylation of p38 MAPK and p44/42 ERK (extracellular-signal-regulated kinase), but inhibition of these kinases alone did not reverse chemotaxis arrest by LPS

**Figure 4:**
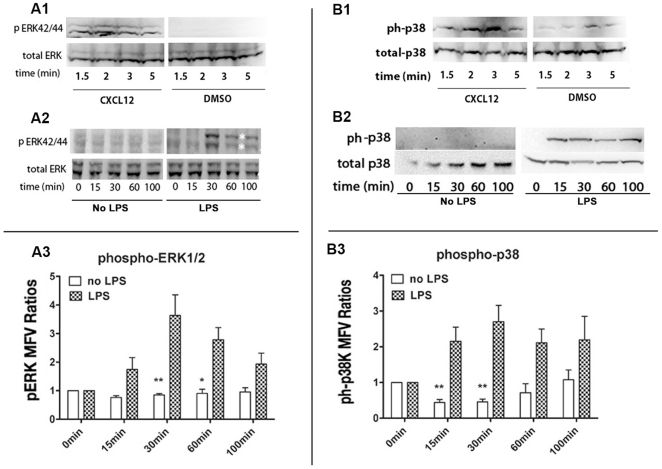
LPS induced phosphorylation of MAPKs. p44/42 ERK (**A1–A3**) and p38 MAPK (**B1–B3**) were phosphorylated after CXCL12 (20 nM) stimulation or LPS (2 ng/ml) treatment. Human monocytes (2×10^6^ cells for each time point) treated with or without CXCL12 were collected at 1.5, 2, 3 and 5 min. Monocytes (5×10^6^ for each time point) treated with or without LPS were collected 0, 15, 30, 60 and 100 min. Half of the cells treated with or without LPS and all the cells treated with or without CXCL12 were extracted with RIPA buffer and proteins were resolved by 4–20% gradient SDS-PAGE. Phospho-ERK and total ERKs were detected by immunoblotting with phospho-ERK specific mAb, E10 and rabbit antibody against total ERK (**A1** and **A2**). Reacting with anti phospho-p38 MAPK (T180/Y182) mAb and rabbit antibody against total p38 detected phospho-p38 and total p38 respectively (**B1** and **B2**). Immunoblots are representative of results with monocytes from 3 donors. The remaining half of the cells treated with or without were stained with a mixture of Alexa-647 E10 mAb against phospho ERK (T202/Y204) and Alexa-488 28B10 mAb against phospho-p38 MAPK (T180/Y182) and analyzed by flow cytometry. Ratios of MFVs for the respective phosho-MAPKs in LPS treated vs. untreated cells are plotted as histograms (n = 5, * p<0.03, ** p<0.01). The remaining cells were extracted with RIPA buffer and proteins resolved by 4–20% gradient SDS-PAGE. Phospho-ERK and total ERKs were detected by immunoblotting with phospho-ERK specific mAb, E10 and rabbit antibody against total ERK. Reacting with anti phospho-p38 MAPK (T180/Y182) mAb and rabbit antibody against total p38 detected phospho-p38 and total p38 respectively (A3 and B3) Immunoblots are representative of results with monocytes from 5 donors.

Since many downstream signaling events of TLR stimulation have been attributed to MAPK activation [4], we evaluated the phosphorylation status of p44/42 ERK and p38 MAPK during LPS treatment, using flow cytometry and immunoblotting. Phosphorylated 44/42 ERK increased significantly after CXCL12 treatment, reaching a maximum at 2 min, then decaying slowly after 5 min of continuous agonist occupancy (Figure 4 A1). By comparison, p44/42 ERK reached a maximum after 30 min LPS treatment, slowly decaying after 30 min (Figure 4 A2 and A3)., Phosphorylation of p38 MAPK followed the same temporal profile as that of p44/42K after CXCL12 treatment (Figure 4 B1). p38 MAPK was phosphorylated after 15 min of LPS treatment, and maintained similar levels of activation up to 100 min after LPS (Figure 4 B2 and B3). Although p38 MAPK phosphorylation by immunoblotting was observed consistently only after 15 min LPS treatment, phosphorylated p38 MAPK immunoreactivity was detected within 5–10 min by flow cytometry (not shown). Since the temporal profile of p38K activation corresponded with that of chemotaxis arrest, we used small molecular weight inhibitors, PD98059 and SB203580 to block activation of p44/42 ERK and p38 MAPK respectively and evaluated monocyte adhesion, chemotactic polarization and migration. Neither kinase inhibitor significantly reversed LPS mediated inhibition of chemotactic polarization or migration towards CCL2 as shown later in the manuscript.

### LPS pretreatment prevented the polarized distribution of activated LFA-1 and Mac-1 in chemokine stimulated monocytes

**Figure 5:**
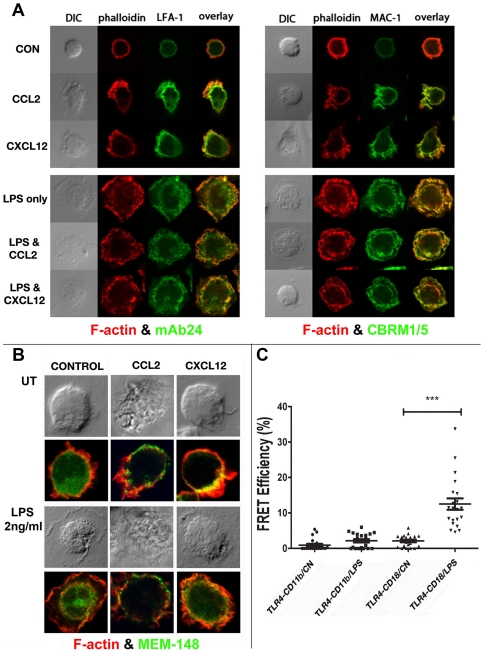
LPS treatment induced global activation of LFA-1 and Mac-1 in human monocytes, which was not reversed by chemokine stimulation. **A**) Human monocytes on cover slips were incubated with (LPS) or without (CON) LPS at 2 ng/ml for 15 min at 37°C. Cells were then washed and treated with CCL2 or CXCL12 (20 nM) for 2 min at 37°C. Cells were fixed, permeablized and stained with phalloidin-568 and murine mAbs against ß2 integrin activation epitopes in LFA-1 (mAb24, left) or Mac-1 (CBRM1/5, right) followed by Alexa-488 conjugated anti-mouse IgG. **B**) Cells treated as described above were stained with phalloidin-568 and mAb MEM148 (followed by Alexa-488 conjugated anti-mouse IgG), which recognizes ß2 integrin activation epitope, irrespective of the heteromeric context. Individual channels corresponding to phalloidin and integrin staining and composite 2-color images are shown along with DIC images. **C**) Acceptor photobleaching FRET assay of proximity interaction between TLR4 and integrins. Human monocytes on cover slips treated with or without LPS (2 ng/ml) for 15 min were fixed and stained with biotin conjugated anti-TLR4 HTA-125 mAb (TLR4) and MEM148 mAb against a β2-integrin activation epitope (CD18) or CBRM1/5 mAb against activated Mac-1 (CD11b), followed by staining with Alexa-488 steptavidin and Alexa-568 goat anti-mouse IgG. FRET was performed in a Leica TCS SP5 confocal microscope. Photo-bleaching was performed with high-intensity laser scanning at 514 nm at 100% power output in defined ROIs (regions of interest). Pre-bleaching and post-bleaching images were acquired, and the average fluorescence intensity of both donor (Alexa-488) and acceptors (Alexa-568) in the bleached ROIs were measured using the proprietary Leica acceptor photobleaching program. E_f_ = (I_post_−I_pre_)/I_pre_ calculated FRET efficiency (E_f_), where *I* corresponds to average intensity of donor (Alexa-488). FRET values averaged from ∼6 ROIs in each of the 6–10 cells from each of 3 separate experiments with different donor cells are plotted in the graph with the mean and standard error drawn as box and whiskers (n = 3, *** p<0.01).

Among the integrins, LFA-1 and Mac-1, which share β2 integrin, respectively with αL and αM subunits play important roles in immune cell migration. Integrin activation exposes hidden epitope(s), and antibodies against these epitope(s) are used as markers of integrin activation Preincubating monocytes with antibodies against (fully extended conformation) LFA-1 (CD11a) or Mac-1 (CD11b) partially inhibited their chemotaxis towards CCL2, CXCL12 or fMLF. However, the blocking antibody L130 against β2 integrin induced an almost complete chemotactic arrest (Figure S4 A). Monocytes treated for 2 min with 20 nM CCL2 or CXCL12 localized activated LFA-1 (Figure 5 A, left) and Mac-1 (Figure 5 A, right) to the leading edge enmeshed within F-actin fibrils whereas in cells pretreated with LPS for 20 min, activated LFA-1, Mac-1 and F-actin were distributed globally (Figure 5 A, bottom panels). We inquired whether LPS induced polarization inhibition could be overcome by higher chemokine levels. We did polarization assays at lower or higher chemokine concentrations. With primary monocytes, optimal polarization was observed after 20 nM CCL2 or CXCL12 for 2 min. At less than 5 nM, F-actin and integrin polarizations were quite modest. 100 nM or higher chemokine levels induced rapid desensitization and signaling decay. LPS pretreatment for 10–15 min abolished integrin polarization induced over a 5–50 nM range of chemokine levels.

Human leukocytes contain a large pool of free β2 integrin, CD18 [22]. A monoclonal antibody, MEM-148, which recognizes free form of β2 integrin induces a high-affinity conformation in the native LFA-1, exposing and binding to its cognate epitope [23]. Mg^++^/EDTA or low pH (5.5–6.5) treatment also exposes the same epitope. Within 15 min of TLR agonist treatment(s), there was a 50% increase in MEM-148 binding, reaching 2.5–3 fold times basal levels after 2–3 h. Among the different TLR ligands, flagellin was the least effective in inducing this conformational change, while ligands for intracellular TLRs (i.e. pI∶C, CpG, poly(U) and muramyl peptide) were ineffectual (Figure S4 B).

MEM-148 also localized to the leading edge of CCL2 or CXCL12 treated monocytes (Figure 5 B). Whether the polarized MEM-18 staining reflected free CD18 or CD18 dissociated from quiescent or activated LFA-1 or Mac-1 heterodimers, LPS blocked β2 integrin localization at the leading edge of CCL2 or CXCL12 treated cells (Figure 5 B). In contrast, CCL2 or CXCL12 treatments did not induce polarized distribution of β1 integrins, visualized by staining with N29 and 21C8 mAbs against β1 integrin heterodimers (data not shown).

We evaluated proximity interaction(s) between activated β2 integrin and the LPS receptor, TLR4 by live FRET microscopy. We measured FRET efficiency on the basis of the increase in the donor fluorescence upon photobleaching the acceptor fluorophore. There was a significant increase in the FRET efficiency between Alexa-568 conjugated MEM-148 mAb recognizing activated β2 integrin (CD18) and Alexa-488 mAb against TLR4 in LPS treated cells, suggesting intimate molecular association between these two proteins. Interaction between activated Mac-1 heterodimer, visualized by CBRM1/5 mAb (CD11b) and TLR4 was not altered by LPS treatment (Figure 5 C). Collectively, these findings suggested that β2 integrin is activated globally at or very close to activated TLR4 sites.

Global integrin activation would be expected to increase cllular adhesion to the substratum and thus counter polarizing chemotactic forces [18], [21]. To address this, we performed a HUVEC (human umbilical vein endothelial cells) cell adhesion assay. We treated monocytes with LPS for 3, 10, 20, 30, 60 min, or 2 h. Alternatively cells were treated with LPS for 30 min, followed by a wash and incubation in LPS free medium for 3, 10, or 120 min. Monocytes were loaded with Calcein A and allowed to adhere to HUVEC monolayer for 30 min. Unbound cells were washed before analysis using Flexstation photometer. As shown in Figure S4 C, increase in the adhesive potential of LPS treated monocytes was inconsistent until after 120 min treatment. However, if the monocytes were rested for 10 min after 30 min LPS treatment resulted in more consistent increase in monocyte adhesion (Figure S4 C). Under our LPS treatment conditions, only the inside-out signaling that leads to partial (and reversible) integrin activation may be operative and may not be sufficient to sustain strong adhesions. In contrast, leukocyte adhesion to inflamed vascular endothelium with chemokines immobilized on the cell surface GAGs (glycoso-aminoglycan) [24] or to LPS treated HUVECs over-expressing ICAM-1 (inter-cellular adhesion molecule) and VCAM-1 (vascular cellular adhesion molecule) [21], [25], [26] are driven by bidirectional signaling that induces fully extended integrin conformations with high-affinity and increased avidity. The inconsistent increase in the adhesion of LPS treated monocytes to untreated HUVECs that we observed may not be sufficient to arrest chemotaxis in the first 15 min of LPS treatment.

### Rap1 is markedly activated and distributed globally in LPS treated monocytes

**Figure 6:**
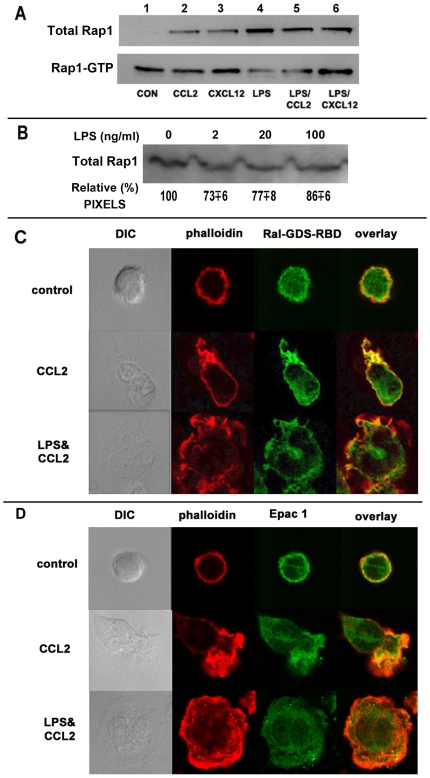
Whereas either chemokine or LPS treatment induced Rap-1 activation in monocytes, LPS treatment disrupted the leading edge localization of activated Rap1 GTPase and Epac-1, the Rap1 GEF. **A**) 10^7^ monocytes were treated with or without LPS (2 ng/ml) for 15 min followed by 2 min treatment with 20 nM CCL2 or CXCL12. Cytoplasmic extracts were prepared and activated (GTP+) Rap1 was isolated using a commercial pull-down assay followed by SDS/PAGE and imunoblotting with anti-Rap1 mAb (upper). Cytoplasmic aliquots were analyzed directly for total cellular Rap1 (lower). **B**) Rap-1 levels were not significantly altered during a 15 min incubation with increasing amounts (2–100 ng/ml) of LPS. Cytoplasmic aliquots were analyzed directly for total cellular Rap1. The relative pixel values for Rap1 represent averaged results from using monocyte from three donors. **C**) LPS treatment abolished polarization of activated Rap1. Human monocytes on cover slips treated with or without LPS (2 ng/ml) for 15 min at 37°C were stimulated with 20 nM CCL2 for 2 min. Cells were fixed, permeabilized and incubated with Ral-GDS RBD GST (Thermo Scientific) for 30 min followed by staining with FITC conjugated anti-GST (BD FACS) and phalloidin-568. **D**) LPS treatment abolished polarization of Rap1 GEF, Epac-1. Monocytes on cover slips treated as above were fixed, permeabilized and incubated with anti-Epac-1 followed by staining with Alexa-488 conjugated anti-rabbit IgG and phalloidin-568. Individual channels corresponding to phalloidin and Rap1 and composite 2-color images are shown along with DIC images. Data are representative of 3 experiments with human monocytes from 3 different donors.

Integrin activation by chemoattractants and inflammatory mediators is mediated by inside out signaling through the small GTPase, Rap1 [19], [27]. Rap1 was activated within 2 min with CCL2 or CXCL12 stimulation (Figure 6 A, lanes 2–3) or after 15 min treatment with LPS alone (Figure 6 A, lane 4), but CCL2 or CXCL12 treatments following LPS did not further enhance Rap1 activation (Figure 6 A, lanes 5–6). Rap-1 levels were not significantly altered during a 15 min incubation with increasing amounts (2–100 ng/ml) of LPS (Figure 6 B). We then examined the sub-cellular distribution of activated Rap1 by reacting with Ral GDS-RBD (Ral guanine nucleotide dissociation stimulator Ras binding domain) GST (glutathione *S*-transferase) fusion protein followed by FITC-conjugated anti-GST antibody. There was significant sequestration of activated Rap1 at the leading edge of cells polarized by CCL2 (Figure 6 C). In cells pretreated with LPS for 15 min, activated Rap1 was globally distributed at the plasma membrane (Figure 6 C). Total Rap1 was distributed all along the peripheral membranes in CCL2 treated cells and in all membranes and focal adhesions in the LPS treated cells (Figure S5 A).

Five different GEFs, namely CalDAG (Ca^++^ and diacyl-glycerol), PDZ GEF1, PDZ GEF2, Epac1 and C3G [28] activate Rap1 under different conditions and in various cell types. Among these GEFs, we found that Epac1 exhibited leading-edge co-localization with F-actin polymers in monocytes polarized by chemoattractant treatment(s); LPS pretreatment induced global recruitment of Epac1 (Figure 6 D).

Since both the release of Epac1 from auto-inhibition and its recruitment to plasma membrane is critically dependent on cAMP (cyclic AMP adenosine (3′-5′) cyclic monophosphate) [29], we inquired whether LPS induced Rap-1 activation followed this pathway. While LPS treatment enhanced cAMP synthesis slightly, this occurred only after 30 min (data not shown). We therefore inquired whether LPS effects could be simulated by treatment with the cAMP analog, 8-(4-chloro-phenylthio)-2-*O*-methyladenosine-3,5-cyclic monophosphate (8CPT-2Me-cAMP) that specifically targets Epac and not PKA [30] and has been shown to induce integrin activation in U937 cells [31]. While 8CPT-2Me-cAMP induced monocyte cell spreading (Fig S5 B) and global Epac1 recruitment, it did not inhibit chemotaxis in primary monocytes (data not shown).

### Simultaneous inhibition of p38 MAPK and Rap1 reversed the LPS induced chemotaxis arrest

**Figure 7:**
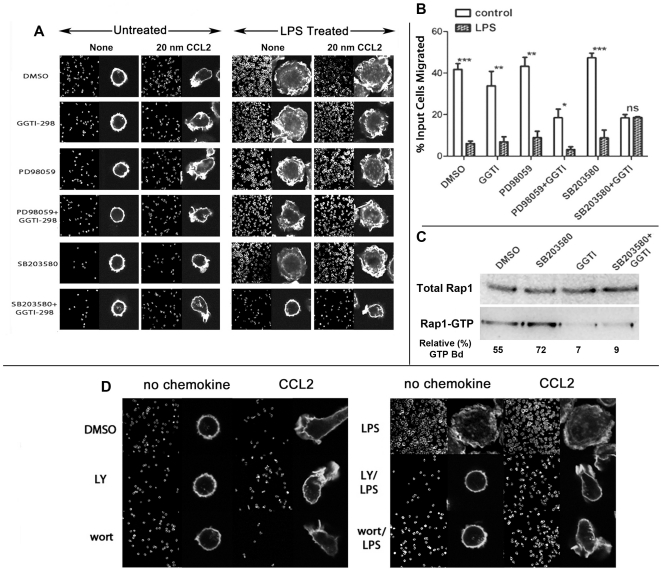
Simultaneous inhibition of Rap1 and p38 MAPK or PI3K inhibition reversed LPS induced block of F-actin polarization and chemotaxis in moncoytes. **A**) Monocytes in RPMI with 5% FBS were treated with 20 µM Rap1 inhibitor GGTI-298 (Calbiochem, EMD Biosci Corp), 10 µM 42/44ERK MAPK inhibitor PD98059 (Cell signaling), 10 µM p38 MAPK inhibitor SB203580 (Tocris Corp), 20 µM GGTI-298+10 µM PD98059 and 20 µM GGTI-298+10 µM SB203580, or DMSO control for 30 min at 37°C. Cells were plated on cover slips and treated with or without LPS (2 ng/ml) at 37°C for 15 min, followed by stimulation with 20 nM CCL2 for 2 min. Cells were fixed and stained with phalloidin-488, and images were collected with Leica TCS SP5. **B**) 10^7^ monocytes were treated with inhibitors as described above for 30 min followed by a 15 min incubation with or without (control) LPS (2 ng/ml). ∼0.5×10^6^ LPS treated or untreated cells in 100 µl of RPMI with 1% FBS were loaded in the upper Transwell chambers (Nunc 5.0 µm) challenged with or without 20 nM CCL2 in the bottom chambers, and incubated at 37°C CO_2_ incubator for 2 h. Chemotaxis was measured as described under Methods. Migrated cells for control and LPS treatments after respective inhibitors are plotted pair wise in the histograms (with error bars). n = 3, ^***^p<0.01,^**^p<0.04, ^*^p<0.05. **C**) LPS induced Rap1 activation was inhibited in monocytes pretreated with GGTI-298 alone or with GGTI-298 and p38 MAPK inhibitor, SB203580, but not with SB203580 alone. Activated (GTP+) Rap1 was extracted from cytoplasmic extracts using a commercial pull-down kit followed by SDS/PAGE and imunoblotting with anti-Rap1 mAb. Numbers represent relative fraction (%) of Rapi that was activated (n = 3). **D**) PI3K inhibitors reversed LPS induced block of F-actin polarization in monocytes stimulated with CCL2. Monocytes in 5%FBS/RPMI were treated with 50 µM LY2940002, 1 µM wortmannin, or DMSO control for 30 min 37°C. Cells were plated on cover slips and treated with or without LPS (2 ng/ml) at 37°C for 20 min, followed by 20 nM CCL2 for 2 min. Cells were fixed and stained with phalloidin-488, and images were collected with Leica TCS SP5.

Rap1 independent pathways may also activate integrins [32]. However since LPS induced marked Rap1 activation, we inquired whether knocking down membrane recruitment and activation of Rap by a specific geranyl transferase inhibitor, GGTI-298 would reverse the chemotaxis arrest by LPS. While GGTI-298 drastically reduced LPS mediated Rap1 activation (Figure 7 C), it did not reverse the cell spreading or the loss of F-actin polarization or trans-well chemotaxis in response to CCL2 in LPS treated cells (Figure 7 A and B).

LPS-induced cell spreading probably reflects unpolarized β2-integrin activation. A linear pathway starting with TLR4/MD1 MyD88 (myeloid differentiation primary response gene 88), IRAK (interleukin-1 receptor-associated kinase), p38 and Rap1 regulates LPS induced β2-integrin activation [19]. Although both MAPK (Fig. 4 A and B) and Rap1 (Figure 6 A) were activated upon LPS simulation, inhibiting either MAPK or Rap1 alone did not reverse the chemotaxis arrest in LPS treated cells (Figure 7 A and B). LPS mediated cell spreading and chemotaxis arrest was reversed only when both Rap1 and p38 MAPK were inhibited with a combination of GGTI-298 and SB203580. In contrast, co-inhibition of Rap1 and ERK (PD98059) was not effective (Figure 7 A and B). We evaluated Rap-1 activation in response to some of these pharmacologic treatments. LPS induced Rap1 activation was inhibited in monocytes pretreated with GGTI alone or with GGTI and p38 MAPK inhibitor, SB203580, but not with SB203580 alone (Figure 7 C). However, combined use of GGTI-298 and SB203580 or PD98059 decreased both chemotactic polarization and migration potential of even the non-LPS treated cells by ∼50%, suggesting that a synergism between MAPK and Rap1 signaling is required for optimal chemotactic response.

### LPS induced rapid global accumulation of PIP_3_ at the monocyte plasma membrane

Consistent with the previous reports [18], [33], we observed that LPS treatment increased PIP_3_ production in monocytes within 3 minutes, which decayed rapidly to basal levels by 5 min (Figure S5 C). In LPS treated cells PIP_3_ was distributed globally at the plasma membrane, unlike CCL2 treated cells where PIP_3_ was localized to the leading edge in the absence of LPS pretreatment (Figure 2A, right). LPS pretreatment abolished the F-actin anisotropy in response to CCL2 (Figure 7 D, right). While PI3K inhibitors LY2940002 and wortmannin did not significantly alter chemotactic polarization in untreated cells (Figure 7 D, left), both inhibitors reversed the LPS induced cell spreading and partially restored chemotactic polarization in response to CCL2 (Figure 7 D, right).

## Discussion

Through multiple criteria, we have shown that LPS and other TLR2/4 ligands induce a rapid arrest of chemotaxis towards CCL2, CXCL12 and fMLF in primary human monocytes by inducing p38 MAPK activation and preventing polarized activation Rap1. This effect was obvious within 15 min of LPS treatment at LPS levels far lower than required for induction of pro-inflammatory cytokine expression. If monocytes were stimulated with CCL2 or CXCL12 before or simultaneously with LPS, chemoattractant signaling is predominant. However, given the protracted nature of TLR signaling and since cells are desensitized rapidly for chemoattractants, cell spreading and chemotactic arrest are the eventual outcome with concomitant chemokine and LPS treatments.

Previous reports have shown that TLR agonists inhibit monocyte chemotaxis through downregulation or degradation CCR2 or other CKRs and/or their mRNAs [12], [13], [14], [15], [16] or by endocytic clearance of receptors from the plasma membrane [17]. Although we observed loss of CCR2 and CXCR4 after prolonged (>1 h) LPS, under more limited treatment conditions, there was no reduction in their steady-state levels or G-protein signaling potential. Furthermore, limited LPS treatment did not enhance, but induced a modest decrease in the endocytosis of agonist occupied CKRs.

Chemokines trigger integrin-dependent leukocyte arrest on the vascular endothelium. Integrin activation harbingers a multi-step adhesion cascade that orchestrates trans-endothelial migration of inflammatory cells [34], [35]. During trans-endothelial migration and subsequent chemotaxis through the parenchyma, leukocytes must execute a delicate balance of adhesion/de-adhesion cycles [34], [35], [36], [37], which is disrupted during pathogen invasion across the vascular barrier.

Through inappropriate and persistent integrin activation LPS induces cell spreading and adhesion, which results in chemotactic arrest [21]. Monocyte adhesion to HUVECs requires LPS treatment at 10 ng/ml for at least 30 min [21], [38], but this was insufficient to block chemotaxis during the first 3–10 min. We have shown that within 2 min of CCL2 or CXCL12 addition, activated integrins are polarized at the leading edge. While LPS and other TLR agonists induced substantial global activation of integrins after 30 min, just 10 min LPS pretreatment was sufficient to subvert the polarized activation of integrin by chemoattractant(s).

Inside out signaling through the small GTPase, Rap1 regulates integrin activation by chemoattractants and other inflammatory mediators [19], [27], [39]. Subsequent signals from activated Rap1 are transduced to RAP1 effector to RapL [40], [41], and then to LFA through SKAP1 [42] and/or Mst1 kinase [43]. In the case of Rap1 activation by LPS or other extracellular stimuli, the Rap1 signal is transduced through distinct effector(s) like RIAM (Rap1–GTP-interacting adapter molecule), KRIT-1/CCM (Krev-*1*/Rap1 interaction trapped 1/cerebral cavernous malformation) and AF-6/Cno (ALL1-fused gene from chromosome 6 protein) proteins, which can assemble adhesion complexes that are distributed uniformly [28].

Of the five different Rap1 GEFs, we found that Epac1 was recruited to the leading edge in chemokine treated monocytes. LPS pretreatment subverted this polarized distribution of Epac1. In both the social ameba, *Dictyostelium* and leukocytes many chemoattractants increase cAMP synthesis through activation of adenylyl cyclase isoforms II, IV, VII and IX by dissociated Gβγ [44] and as such, cAMP synthesis was relatively enriched at the leading edge of the polarized leukocyte. We found that LPS treatment slightly enhanced cAMP synthesis (data not shown), which may lead to global Epac1 recruitment.

Rap1 independent pathways also activate integrins [32]. For instance, LPS at 1 µg/ml increased adherence of THP-1 monocytes to immobilized sICAM-1, through PI3K mediated LFA-1 activation [18]; or through actin reorganization by activated proline-rich tyrosine kinase 2 (Pyk2) and paxillin [20]; or through a signaling pathway via MyD88, IRAK, p38, and Rap1 [19]. Consistent with these reports, we found that PI3K inhibitors reversed LPS induced cell spreading and restored the chemotactic polarization in response to CCL2.

However, PI3K inhibitors did not block the CCL2 mediated F-actin polarization in monocytes. Of the multiple isoforms of PI3K, denoted as Class Ia, Ib, II and III [45], only PI3Kδ a Class Ia member, and PI3Kγ, the sole Ib enzyme, have central roles in leukocyte chemotaxis [46], [47], [48]. PI3Kγ is activated by the Gβγ subunits dissociated from the agonist bound GPCR [45], whereas Class Ia enzyme(s) are activated by binding to phospho-tyrosine motifs on receptors utilizing protein tyrosine kinases as their proximal signal transduction element [49], [50] as during TLR signaling. General PI3K inhibitors, such as wortmannin or LY294002 may not have knocked out the different PI3K isoforms equally well. Alternatively, the failure of PI3K inhibitors may reflect alternative regulation of chemotaxis by PLA2 and PLC (phospholipase A2 and C) [51], [52], [53]. PI3K itself is regulated through PIP2/PTEN by the PLC pathway, while PLA2 depends on cytosolic Ca^++^, which is regulated by IP3 (and thus indirectly by PLC), fatty acids (and thus partly by PLA2), and Ca^++^ uptake.

An intracellular signaling hierarchy regulates neutrophil migration in opposing gradients of intermediate vs. end-stage chemoattractants (i.e.CXCL8 vs. fMLF) that use PI3K or p38 MAPK pathways respectively [54], [55]. Furthermore, LPS was shown both *in vitro* and *in vivo* to be a p38 MAPK dependent disrupter of neutrophil migration towards intermediate chemoattractants [11], however the corresponding mechanisms are not as well defined for monocytes. PI3K/Akt pathway has been reported to be able to, in turn negatively regulate TLR signaling pathways at different steps including MAPK cascades (JNK, p38, ERK) and NF-κB signaling network [56], [57].

We confirmed the previous leads showing that LPS induced modulation of monocyte and neutrophil chemotaxis was dependent on p44/42 ERK and/or p38 MAPK signaling [11], [54], [58], [59], While small molecular weight inhibitors against MAPKs were unsuccessful in reversing the LPS effect on monocytes, a partial reversal of LPS effect was achieved monocytes through the combined inhibition of Rap1 and p38 MAPK with the caveat that chemotaxis of untreated cells was also decreased by these agents.

Integrin activation by chemoattractants and other inflammatory mediators is mediated by inside out signaling through Rap1 [19], [27], [39]. Polarized Rap1 activation helps reinforce the leading edge by providing focal nodes for integrin activation and F-actin attachment. While global Rap1 activation in LPS treated cells would subvert the nascent leading edge, leading to cell spreading, inhibiting Rap1 *alone* may not be sufficient to reverse the LPS effect through other p38 MAPK targets. Inhibiting both Rap1 and p38 MAPK would be expected to have a cumulative suppressive effect on cell polarization and chemotactic potential. Since RAP1 and p38 MAPK activation constitute the earliest cellular response to LPS treatment, inhibiting both of them eliminates LPS effect on chemotactic polarization.

It was of interest that neutrophil polarization and chemotaxis were unaffected by TLR ligands under conditions used for monocytes. However, neutrophils were just as competent as monocytes in setting up pro-inflammatory cytokine expression after TLR stimulation. Neutrophils have a short lifespan and are more proficient sentinels of pathogen invasion than monocytes. It is therefore reasonable that they have a higher set point for chemotactic arrest by LPS or other TLR ligands. Although they eventually succumb to LPS mediated arrest, they are endowed with a robust PI3K/PTEN system [60], [61], [62] that preserves chemotactic polarization under the more limited LPS treatment conditions. Neutrophils are also relatively deficient in some key Rap-1 activators such as Epac1, which is recruited to the plasma membrane by cAMP [31]; instead they prefer CalDAG GEF, which is recruited by products of PIP_2_ hydrolysis by Gβγ activated PLC-β [63]. If and when neutrophils reach the pathogen zone, the high levels of PAMPs can initiate a robust TLR signaling leading to chemotactic arrest and formation of neutrophil extracellular traps (NETs) [64], [65].

Blood monocytes are a heterogeneous population and in humans three different populations have been identified on the basis of CD14 and CD16 expression [66] and two major human monocyte subsets, the CD14^+^CD16^−^ and CD14^lo^CD16^−^ monocytes are defined by CX_3_CR1 expression [67]. CD14^+^CD16^−^ monocytes, which constitute about 90% of fresh blood monocytes were arrested for chemotaxis by LPS treatment. On plating overnight, CD14^lo^CD16^−^ and CD14^+^CD16^+^ species emerge, which preserved LPS induced chemotaxis arrest (not shown) [55].

Monocytes represent a systemic reservoir of relatively immature myeloid precursors, which differentiate to macrophages DCs (dendritic cells) and other APCs depending upon the cytokine environment [68], [69], [70]. The chemotactic arrest monocytes experience upon entering the pathogen milieu is consistent with their rear guard role against pathogen invasion, both by way of enhanced phagocytosis (Figure S5 C) and antigen processing. Exposure to low level LPS and other bacterial antigens may influence monocyte differentiation potential into macrophsges or DCs and thus modulate the induction of adaptive immune responses to infection [71]. Stimulating monocytes simultaneously with multiple TLR ligands as during natural infection may enhance chemokine secretion, and thus overcome the temporary inhibition of chemotaxis. By the same token, multiple bacterial ligands may impose more lasting adhesion and chemotaxis inhibition. However, the relative magnitudes of these phenotypes can only be evaluated in sepsis or during massive experimentally induced peritonitis. Our model is still valid for most natural (pre-clinical) infections where neutrophils are active patrollers and monocytes and DCs initiate the transition from innate to acquired immunity.

## Materials and Methods

### Cells

Elutriated human monocytes of >98% purity (as judged by CD14 receptor density) was from the Department of Transfusion Medicine at the NIH Clinical Center. Human neutrophils were purified from whole blood as described [72]. HUVECs and U937 cells were purchased from ATCC.

### Biochemicals and Reagent Kits

Lipopolysacharide (LPS, from E.coli, serotype O55∶B5 or R515 (Re)), lipid A (from E.coli, Serotype R515 (Re)), Macrophage stimulatory lipopeptide 2 (MALP2), Flagellin, poly (I∶C), poly U, CpG OND, Pam3CSK4, Ac-muramyl-Ala-D-Glu-amide were purchased from Alexis Biochemicals. Human CCL2, CXCL8, CXCL12 and fMLF were purchased from Biosource; FURA-2 AM for calcium signaling and phalloidin-568 or phalloidin-488 from Invitrogen. Active Rap1, Rac1, CDC42, pull-down and detection assay kits and Rap1 assay reagent (Ral GDS-RBD-GST) were from Piercenet subsidiary of Thermo Scientific; human cytokine test kits for use in the Luminex plate reader were from Millipore; p38 kinase inhibitor, SB203580, ERK inhibitor, PD98059; PI3K inhibitors, LY2940002 and wortmannin; and Rap-1 specific geranyl-geranyl-transferase inhibitor, GGTI-298 were bought from Calbiochem, EMD Biosciences. 8CPT-2ME-cAMP was from Tocris Corp.

### Antibodies

Unconjugated or Alexa dye conjugated murine mAbs against phospho-Akt (S473), phospho-p44/42 MAPK (T202/Y204) (clone E10), phospho-p38 (T180/Y182) (clone 28B10) were from Cell Signaling; unconjugated or biotinylated anti-PIP_3_ IgM mAb was from Echelon Corp. Murine anti-CD11a, anti-CD11b, anti-CD18 (L130) mAbs were from BD scientific; unconjugated or Alexa dye conjugated antibodies against CD4, CCR2A/2B, CCR3, CCR5, CCR7, CXCR-1, 2, 3 and 4 were purchased from R&D systems, MN or BD Biosciences, CA.; murine mAb, CMBR1/5 against activated Mac-1 was from eBiosciences; N29 and 21C8 mAbs against activated β1 integrin heterodimers and CBRM1/5 mAb against activated β2 integrin in Mac-1and rabbit mAbs against Rap1 and Epac1 were from Abcam. Murine mAb against activated LFA-1 (mAb24) was kindly provided by Dr. Nancy Hogg of Cancer Research UK.

### TLR Agonist treatments

TLR agonists were used at the following concentration unless otherwise specified: LPS, 2 ng/ml, Poly (I∶C), 20 µg/ml, MALP-2, 200 ng/ml, CpG OND, 2 µg/ml, Pam3CSK4, 50 ng/ml, poly U, 1 µg/ml, flagellin, 200 ng/ml, muramyl-Ala-D-Glu-amide, 100 ng/ml, lipid A, 200 ng/ml. Human primary monocytes or neutrophils were collected and diluted at 10×10^6^ cells per ml for the each respective TLRs agonist stimulation at 37°C in 5% CO_2_ incubator for the indicated duration. In every experiment (unless specifically indicated otherwise), cells were washed 3X with 5 volumes of RPMI (with 5% FBS) before assays.

### Immunoblotting

Treated human monocytes or neutrophils lysed with RIPA buffer (50 mM HEPES 7.0, 150 mM NaCl, 0.5% Triton X-100, 10% glycerol and protein inhibitor cocktail) at RT for 10 min, lysates was cleared by at 13,000 rpm spin for 10 min, supernatant was collected, mixed with 4 X LAD buffer containing 2 mM DTT (Invitrogen) and boiled for 5 min before SDS/PAGE (4/20%) and transfer to PVDF or nitrocellulose membranes (Invitrogen) and immunoblotted with various antibodies followed with HRP conjugated anti-mouse or anti-rabbit second antibody (Thermo Scientific). Stained protein bands were developed with Supersignal west Femto maximum sensitivity substrates (Thermo Scientific)

### Flow Cytometry

Cell surface expression of various receptors and intracellular signaling molecules and F-actin were tested by staining with fluorescent reagents, and analyzed by flow cytometry as described extensively before [72]. Monocytes stimulated for 30 min with 100 nM PMA, ionomycin (0.5 µg/ml) or both were used as positive controls for measuring intracellular kinases and other signaling molecules. Data were analyzed with program FlowJo software, version 9.2.

### Endocytosis assay

FACS based quantitative analysis of receptor density and receptor internalization and microscopic visualization of agonist driven endocytosis have been described [73]. In some experiments cell surface bound antibody was stripped by treatment for 2 min with 0.5% acetic acid in 500 mM NaCl.

### Intracellular (Ca^++^) flux

For intracellular Ca^2+^ flux in 2×10**^6^** monocytes were loaded with Fura-2 AM in for 45 min at 37°C HBSS with Ca^2+^ and Mg^2+^ and containing 1% (w/v) dextrose and 0.5% (w/v) of highly purified BSA. Cells were washed and resuspended at 1×10**^6^**/ml prior to sequential agonist stimulation in a continuously stirred cuvette at 37°C in a fluorimeter (Photon Technology Inc., South Brunswick, NJ as described. Where indicated, cells were treated with LPS before agonist treatment. Data were recorded every 200 ms as the relative ratio of fluorescence emitted at 510 nm after sequential excitation at 340 and 380 nm. The integrity of assay was assessed by comparing the signaling response to ionomycin treatment before and after each run and the metabolic integrity of neutrophils was evaluated by comparing the Ca^2+^ flux in response to ATP before and after each run [73].

### cAMP assay

Intracellular cAMP levels were assayed using direct cAMP Enzyme Immunoassay kit (Assay Designs) following manufacturer's instructions. The optical density was read at 405 nm in FlexStation II microplate scanner and the results were analyzed using Softmax Pro5 (Molecular Devices,Sunnyvale, CA).

### Chemotaxis Assays

End-point chemotaxis was determined using the Trans-well system with membranes of 6.5 mm diameter and 5.0 µ pore size in RPMI containing 10 mM HEPES and 1% FBS as described [73]. Cells were incubated for 1.5–2 h for neutrophils and 2–2.5 hrs for monocytes. The ratios of migrated cells were determined from the number of cells in the lower and upper chambers counted in a cell sorter after addition of a known number of fluorescent reference particles (Spherotech, Inc. Libertyville, IL) [72].

### Immunofluorescence Microscopy

Cells plated on cover slips after respective treatments were fixed, permeablized and stained with fluorescent antibodies. Cells were processed for microscopy as described previously [72]. To −15 -visualize intracellular lipid and protein mediators of chemotactic signaling phalloidin-stained cells were reacted with antibodies against the respective targets [72] in PBS containing saponin (0.5 mg/ml). Cells were counterstained with dye-conjugated secondary antibodies as indicated. Cells were visualized by confocal microscopy as described [74] using a Leica TCS-NT/SP5 confocal microscope (Leica, Exton, PA USA) equipped with 100X oil immersion objective NA 1.32. Images were processed using the Leica TCS-NT/SP2 software (version 2.1347) Image-J (version 1.42l), and Adobe Photoshop CS4.

## Supporting Information

Figure S1 Chemotaxis inhibition occurred early after TLR2/4/6 agonist treatment and was generally irreversible. Monocytes in triplicate were treated at 37°C with LPS (2 ng/ml) or MALP-2 (200 ng/ml) for 3, 10, 20, 60 and 180 min, washed twice and allowed to migrate towards 20 nM CCL2 or CXCL12 in a Transwell chamber. Alternatively, cells were treated with the TLR agonists for 20 min and then washed and rested for 3, 10, 20, 60 or 120 min (A3–A120) before chemotaxis in Transwell chambers. Data are plotted as histograms with error bars (n = 3).
(TIF)

Figure S2 Cytokine and chemokine secretion profiles of monocytes treated with selected TLR ligands. Fresh monocytes were treated with TLR 2 (Pam_3_CSK_4_) or TLR4 (LPS) ligands for the indicated times and cell supernatants were analyzed for cytokine production using Luminex cytokine profiling assay. **A**) Normalized values (in pg/ml) of different cyto/chemokines are tabulated for each treatment. **B**) Average cyto/chemokine values from two experiments for a limited set of TLR ligand treatments are plotted as histograms.
(TIF)

Figure S3 Short term LPS LPS treatment did not enhance the magnitude of internalization of agonist occupied CCR2 and CXCR4, but rather induced a modest inhibition of receptor clearance at the higher agonist inputs. LPS (2 ng/ml for 15 min at 37°C) treated or untreated monocytes (10^7^ cells/ml) were stimulated with increasing amounts of CCL2 or CXCL12 for 15 min (left), or were stimulated with 100 nM of CCL2 or CXCL12 for various times (right). Cell surface receptor densities were evaluated by FACS analysis and data are presented as percent of MFV values before agonist binding. Data from four different donors were fit to polynomial regression curves with error bars. EC_50_ and *t*_1/2_ values for the respective CKR/CK combinations are denoted by the interpolations on the *abscissa*.
(TIF)

Figure S4 **A**) Selected antibodies against quiescent or activated heterodimers inhibit relative chemotaxis of monocytes towards CCL2, CXCL12 or fMLF. after preincubation with the indicated antibodies. Fresh monocytes (10^7^/ml) in RPMI (5% FCS) medium pre-treated for 15 min with the indicated antibodies or IgG control prior to chemotaxis assay in Transwell chambers against the indicated agonists. Fraction of migrated cells for each condition is pltted in the histogram (with error bars, n = 4). **B**) Time course of ß-2 integrin activation by TLR agonists. Fresh monocytes (10^7^/ml) in RPMI (5% FCS) were treated with the indicated TLR or NOD2 ligands and 100 µl samples were collected after 0,15, 30, 60, 100, 180 min or overnight (16 hrs) treatment and stained with APC conjugated MEM148 mAb targeted against the ß-2 integrin activation epitope at 4°C for 30 min, and analyzed by flow cytometry. MFVs relative to untreated cells are plotted as histograms (with error bars) (n = 3). **C**) Time course of monocyte adhesion after LPS stimulation, HUVEC (ATCC, PCS-100-030, second passage) cells were incubated in sterile Greiner black transparent 96-well plates at density of 30000 cells/well one day before the assay. Monocytes treated with DMSO or LPS (2 ng/ml) for 3, 10, 60 or 120 min were washed 3 X and resuspended at 5×10^6^ cells/ml in RPMI without serum and containing 2.5 µM calcein AM at 37°C for 30 min. Cells were washed thrice and resuspended in RPMI at 2.5×10^6^ cells/ml and 100 µl of calcein-labeled cells were layered on HUVEC cells in 96-well plates and incubated for 30 min.. Non-adherent cells were removed by 5 X washes and calcein fluorescence was measured by Flexstation at Ex494/Em517. Cell adhesion (%) was determined from the fraction of bound to total fluorescence. Alternatively, monocytes were rested for 3 (A3), 10 (A10) or 120 min (A120) in normal medium after 30 min pre-treatment DMSO (clear bars) or LPS (2 ng/ml) (black bars), before loading and HUVEC adhesion. Histograms are representative of two experiments.
(TIF)

Figure S5 **A**) LPS pretreatment abolished polarized Rap1 accumulation in monocytes following chemokine stimulation. Monocytes (10^6^) on cover slips were treated with or without LPS (2 ng/ml) for 15 min at 37°C and then stimulated with 20 nM CCL2 or CXCL12 for 2 min. Cells were fixed, permeabilized and incubated with rabbit anti-Rap1 antibody followed by Alexa488-anti-Rabbit IgG. Individual channels corresponding to phalloidin and Rap1 and composite 2-color images are shown along with DIC images. Data are representative of 3 experiments with human monocytes from 3 different donors. **B**) Epac-1 activation by 8CPT-2Me-cAMP induced cell-spreading resembling that induced by LPS treatment. Monocytes (0.5×10^6^) were treated with DMSO or LPS (2 ng/ml) at 37 degrees for 15 min or 8CPT-2Me-cAMP (400 µM) for 40 min in 100 µl of RPMI containing 1% FBS. Cells were fixed and stained with Alexa-568 conjugated phalloidin and examined by fluorescent microscopy. Photomicrograph is representative of three independent experiments using cells from three donors. **C**) LPS treatment induced rapid PIP_3_ accumulation, which decayed just as quickly. Monocytes (∼10^6^ cells), treated with or without LPS (2 ng/ml) for 1, 2, 3, 4, 5 or 30 min were collected, fixed, and permeabilized with 0.2% saponin for 10 min at 4°C, incubated with mouse anti-PIP_3_ IgM (Echelon) followed by staining with Alexa-488 anti-mouse IgM for 30 min and analyzed by flow cytometry. Histogram (with error bars) shows PIP_3_ MFVs averaged from three experiments (n = 3).
(TIF)
