# Supplementary figures and images for: TLR Signaling Paralyzes Monocyte Chemotaxis through Synergized Effects of p38 MAPK and Global Rap-1 Activation
Source: PLoS One. 2012 Feb 9;7(2):e30404. doi: 10.1371/journal.pone.0030404 (PMC3276499; doi:10.1371/journal.pone.0030404)

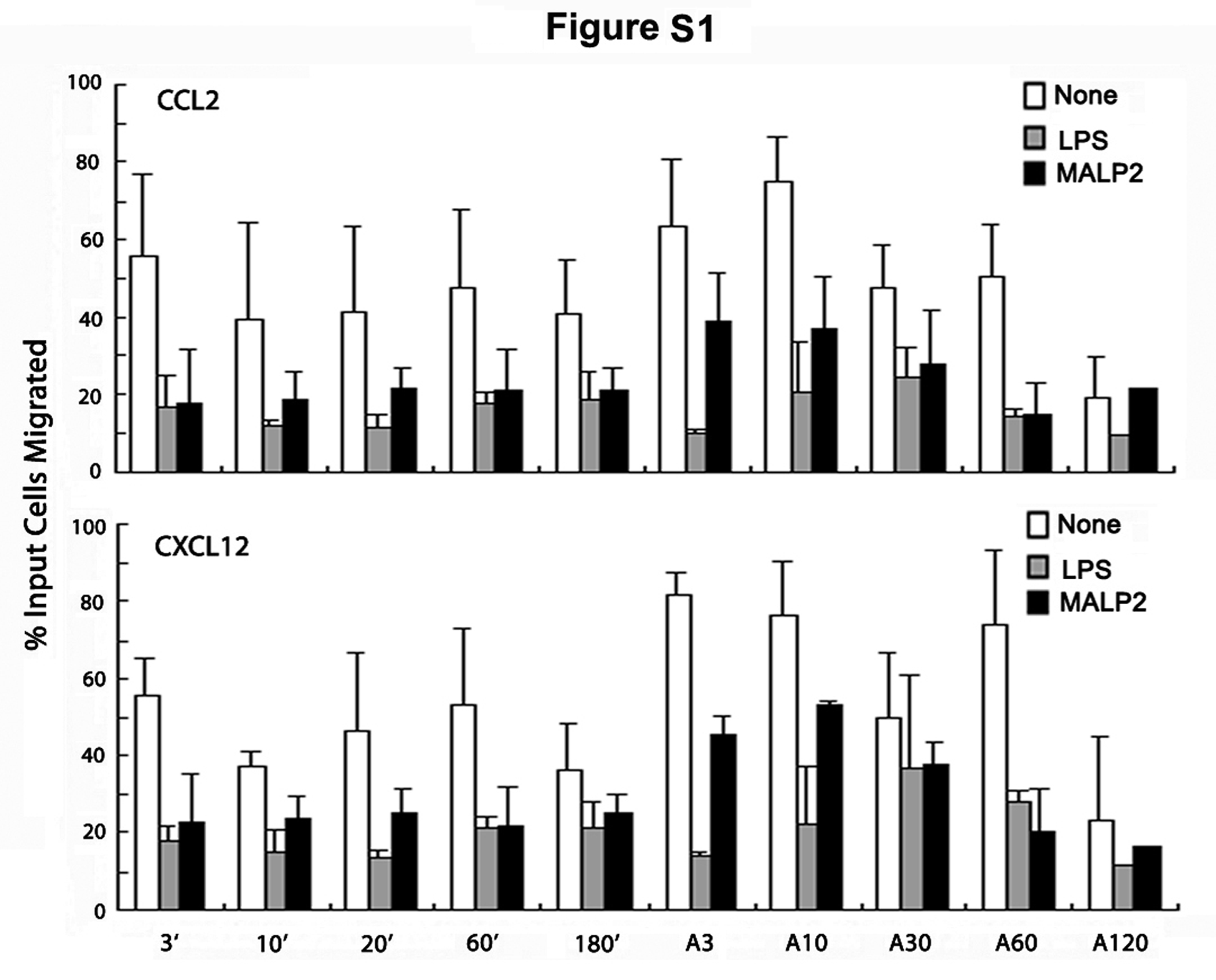

Supplement: Figure S1 — Chemotaxis inhibition occurred early after TLR2/4/6 agonist treatment and was generally irreversible. Monocytes in triplicate were treated at 37°C with LPS (2 ng/ml) or MALP-2 (200 ng/ml) for 3, 10, 20, 60 and 180 min, washed twice and allowed to migrate towards 20 nM CCL2 or CXCL12 in a Transwell chamber. Alternatively, cells were treated with the TLR agonists for 20 min and then washed and rested for 3, 10, 20, 60 or 120 min (A3–A120) before chemotaxis in Transwell chambers. Data are plotted as histograms with error bars (n = 3). (TIF) [file pone.0030404.s001.tif]

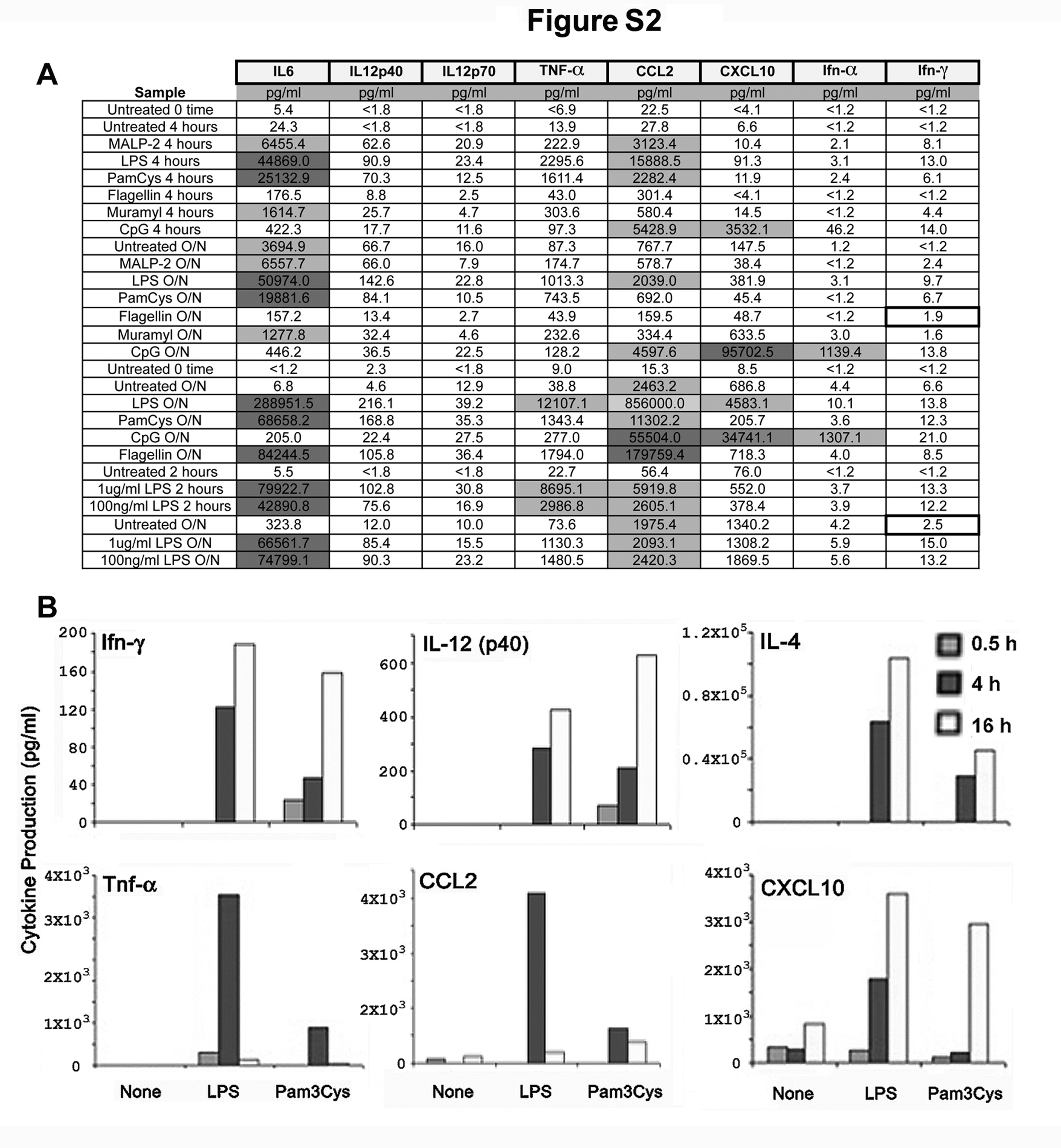

Supplement: Figure S2 — Cytokine and chemokine secretion profiles of monocytes treated with selected TLR ligands. Fresh monocytes were treated with TLR 2 (Pam3CSK4) or TLR4 (LPS) ligands for the indicated times and cell supernatants were analyzed for cytokine production using Luminex cytokine profiling assay. A) Normalized values (in pg/ml) of different cyto/chemokines are tabulated for each treatment. B) Average cyto/chemokine values from two experiments for a limited set of TLR ligand treatments are plotted as histograms. (TIF) [file pone.0030404.s002.tif]

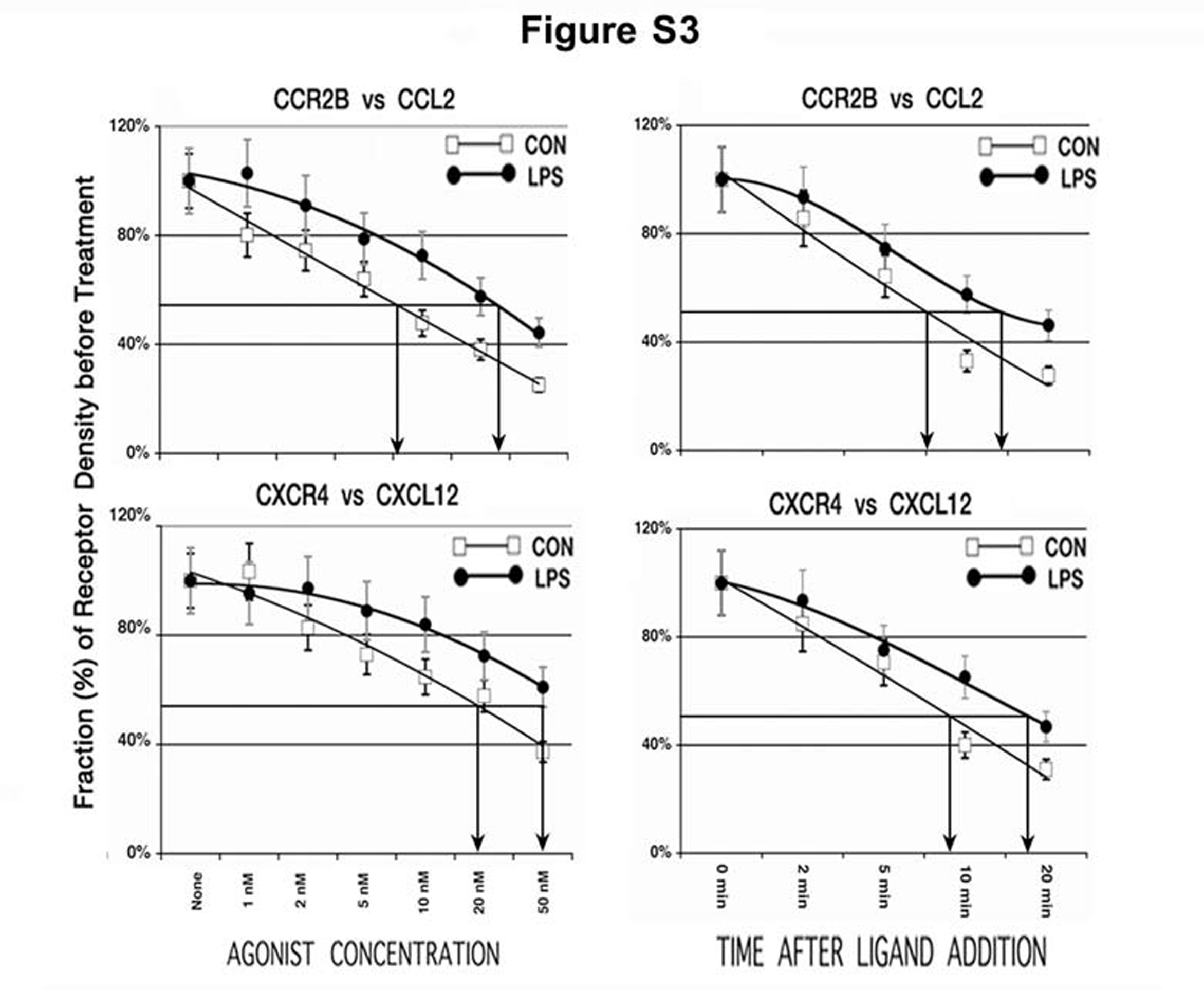

Supplement: Figure S3 — Short term LPS LPS treatment did not enhance the magnitude of internalization of agonist occupied CCR2 and CXCR4, but rather induced a modest inhibition of receptor clearance at the higher agonist inputs. LPS (2 ng/ml for 15 min at 37°C) treated or untreated monocytes (107 cells/ml) were stimulated with increasing amounts of CCL2 or CXCL12 for 15 min (left), or were stimulated with 100 nM of CCL2 or CXCL12 for various times (right). Cell surface receptor densities were evaluated by FACS analysis and data are presented as percent of MFV values before agonist binding. Data from four different donors were fit to polynomial regression curves with error bars. EC50 and t 1/2 values for the respective CKR/CK combinations are denoted by the interpolations on the abscissa. (TIF) [file pone.0030404.s003.tif]

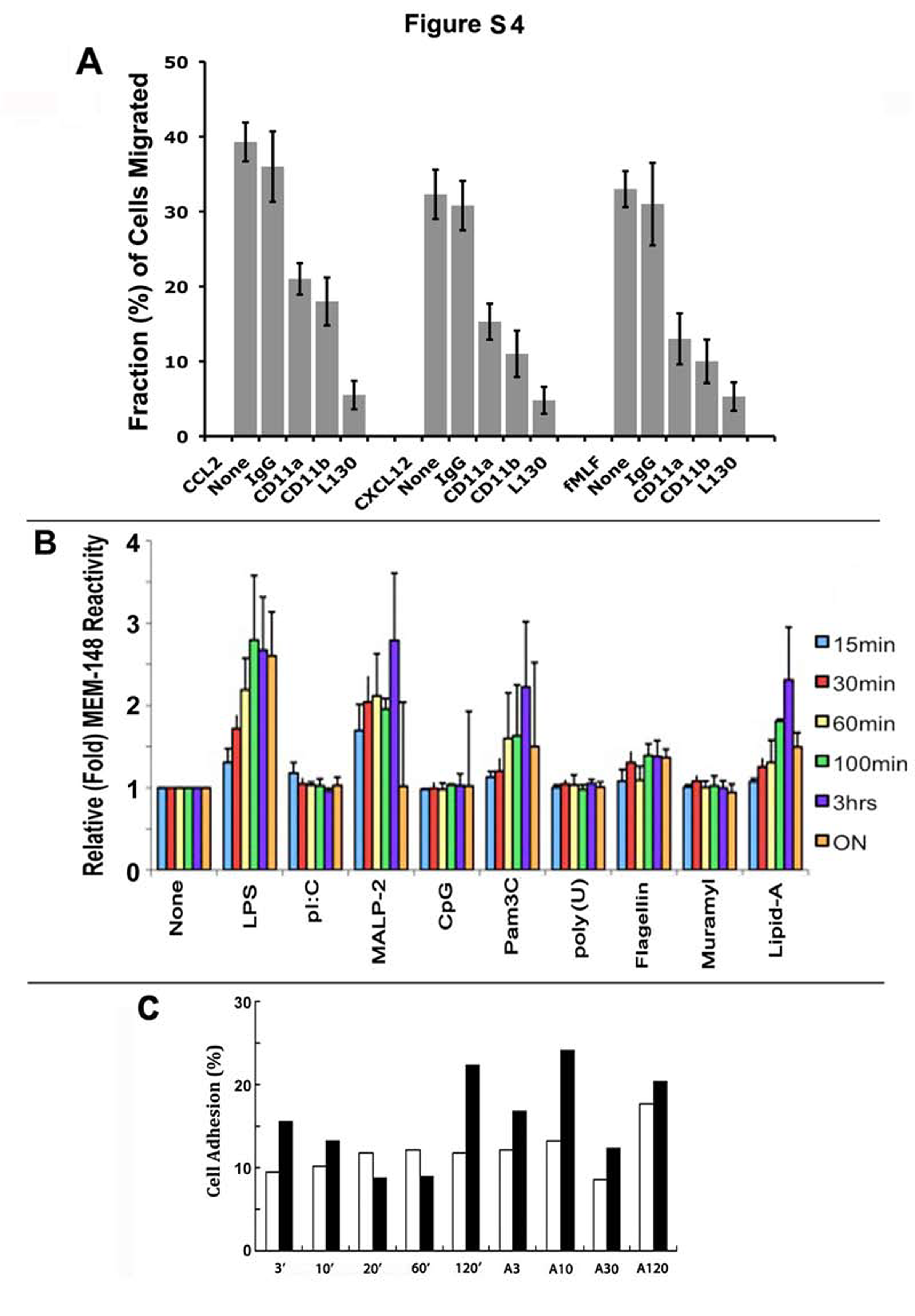

Supplement: Figure S4 — A) Selected antibodies against quiescent or activated heterodimers inhibit relative chemotaxis of monocytes towards CCL2, CXCL12 or fMLF. after preincubation with the indicated antibodies. Fresh monocytes (107/ml) in RPMI (5% FCS) medium pre-treated for 15 min with the indicated antibodies or IgG control prior to chemotaxis assay in Transwell chambers against the indicated agonists. Fraction of migrated cells for each condition is pltted in the histogram (with error bars, n = 4). B) Time course of ß-2 integrin activation by TLR agonists. Fresh monocytes (107/ml) in RPMI (5% FCS) were treated with the indicated TLR or NOD2 ligands and 100 µl samples were collected after 0,15, 30, 60, 100, 180 min or overnight (16 hrs) treatment and stained with APC conjugated MEM148 mAb targeted against the ß-2 integrin activation epitope at 4°C for 30 min, and analyzed by flow cytometry. MFVs relative to untreated cells are plotted as histograms (with error bars) (n = 3). C) Time course of monocyte adhesion after LPS stimulation, HUVEC (ATCC, PCS-100-030, second passage) cells were incubated in sterile Greiner black transparent 96-well plates at density of 30000 cells/well one day before the assay. Monocytes treated with DMSO or LPS (2 ng/ml) for 3, 10, 60 or 120 min were washed 3 X and resuspended at 5×10^6^ cells/ml in RPMI without serum and containing 2.5 µM calcein AM at 37°C for 30 min. Cells were washed thrice and resuspended in RPMI at 2.5×10^6^ cells/ml and 100 µl of calcein-labeled cells were layered on HUVEC cells in 96-well plates and incubated for 30 min.. Non-adherent cells were removed by 5 X washes and calcein fluorescence was measured by Flexstation at Ex494/Em517. Cell adhesion (%) was determined from the fraction of bound to total fluorescence. Alternatively, monocytes were rested for 3 (A3), 10 (A10) or 120 min (A120) in normal medium after 30 min pre-treatment DMSO (clear bars) or LPS (2 ng/ml) (black bars), before loading and HUVEC adhesion. Histograms are represent [file pone.0030404.s004.tif]

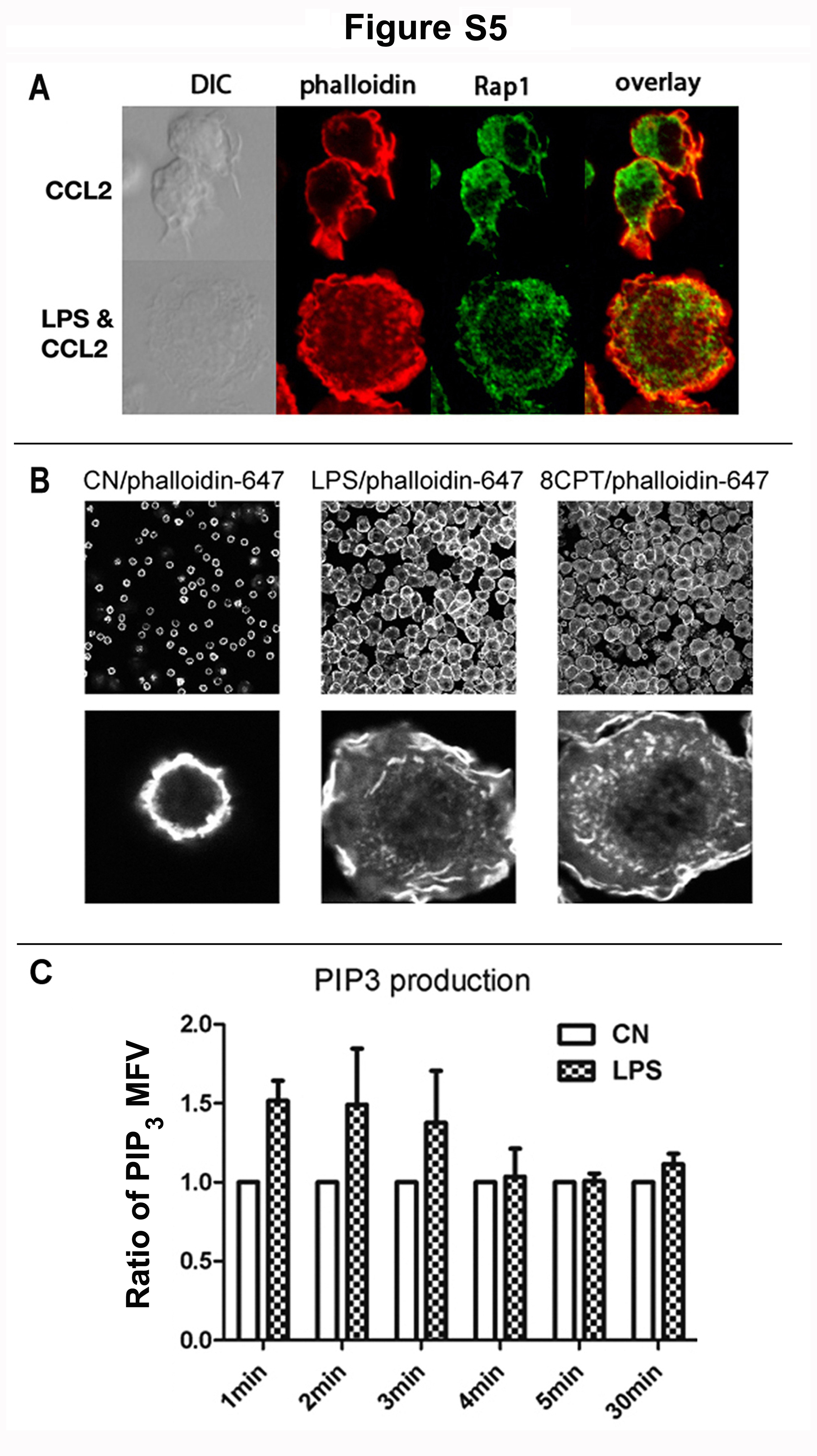

Supplement: Figure S5 — A) LPS pretreatment abolished polarized Rap1 accumulation in monocytes following chemokine stimulation. Monocytes (106) on cover slips were treated with or without LPS (2 ng/ml) for 15 min at 37°C and then stimulated with 20 nM CCL2 or CXCL12 for 2 min. Cells were fixed, permeabilized and incubated with rabbit anti-Rap1 antibody followed by Alexa488-anti-Rabbit IgG. Individual channels corresponding to phalloidin and Rap1 and composite 2-color images are shown along with DIC images. Data are representative of 3 experiments with human monocytes from 3 different donors. B) Epac-1 activation by 8CPT-2Me-cAMP induced cell-spreading resembling that induced by LPS treatment. Monocytes (0.5×10^6^) were treated with DMSO or LPS (2 ng/ml) at 37 degrees for 15 min or 8CPT-2Me-cAMP (400 µM) for 40 min in 100 µl of RPMI containing 1% FBS. Cells were fixed and stained with Alexa-568 conjugated phalloidin and examined by fluorescent microscopy. Photomicrograph is representative of three independent experiments using cells from three donors. C) LPS treatment induced rapid PIP3 accumulation, which decayed just as quickly. Monocytes (∼106 cells), treated with or without LPS (2 ng/ml) for 1, 2, 3, 4, 5 or 30 min were collected, fixed, and permeabilized with 0.2% saponin for 10 min at 4°C, incubated with mouse anti-PIP3 IgM (Echelon) followed by staining with Alexa-488 anti-mouse IgM for 30 min and analyzed by flow cytometry. Histogram (with error bars) shows PIP3 MFVs averaged from three experiments (n = 3). (TIF) [file pone.0030404.s005.tif]
